# Analysis of Trace Metals in Human Hair by Laser-Induced Breakdown Spectroscopy with a Compact Microchip Laser

**DOI:** 10.3390/s21113752

**Published:** 2021-05-28

**Authors:** Makoto Nakagawa, Yuji Matsuura

**Affiliations:** 1 Graduate School of Engineering, Tohoku University, 6-6-05 Aoba, Sendai 980-8579, Japan; makoto.nakagawa.p1@dc.tohoku.ac.jp; 2 Graduate School of Biomedical Engineering, Tohoku University, 6-6-05 Aoba, Sendai 980-8579, Japan

**Keywords:** laser-induced plasma spectroscopy, microchip laser, hair analysis

## Abstract

A laser-induced breakdown spectroscopy (LIBS) system using a microchip laser for plasma generation is proposed for in-situ analysis of trace minerals in human hair. The LIBS system is more compact and less expensive than conventional LIBS systems, which use flashlamp-excited Q-switched Nd:YAG lasers. Focusing optics were optimized using a Galilean beam expander to compensate for the low emitted pulse energy of the microchip laser. Additionally, hundreds of generated LIBS spectra were accumulated to improve the signal-to-noise ratio of the measurement system, and argon gas was injected at the irradiation point to enhance plasma intensity. LIBS spectra of human hair in the UV to near IR regions were investigated. Relative mass concentrations of Ca, Mg, and Zn were analyzed in hairs obtained from five subjects using the intensity of C as a reference. The results coincide well with those measured via inductively coupled argon plasma mass spectrometry. The lowest detectable concentrations of the measured LIBS spectra were 9.0 ppm for Mg, 27 ppm for Zn, and 710 ppm for Ca. From these results, we find that the proposed LIBS system based on a microchip laser is feasible for the analysis of trace minerals in human hair.

## 1. Introduction

Laser-induced breakdown spectroscopy (LIBS) is a technique that measures emission spectra from luminous plasma generated by irradiation with nano-, pico-, and femto-second laser pulses and is useful for multi-elemental analysis of various target materials [1,2]. A microchip laser [3,4,5,6] that emits a pulse energy of hundreds of microjoules has become popular because it makes the LIBS system more compact and lower in cost than conventional systems, which use flashlamp-excited Q-switched Nd:YAG lasers [7,8]. Portable LIBS systems with microchip lasers and compact fiber-coupled spectrometers have been developed [9]. These compact systems have been used for quantitative analysis of steel composition [10,11] and aluminum alloys [12,13].

LIBS techniques are useful for qualitative and quantitative analysis of biological samples and have been applied in the diagnosis of some diseases, such as cancer [14]. In biomedical applications, one of the advantages of the LIBS technique is that pretreatment of samples is not required, unlike in other elemental analysis methods such as inductively coupled argon plasma-atomic emission spectroscopy (ICP-AES) or mass spectrometry (ICP-MS) [15]. For healthcare applications, such as nutritional status monitoring, analysis of easily harvested biological specimens, such as nails and hair, is useful. ICP-AES and ICP-MS have already been applied to the analysis of a variety of biological samples, including nails and hair [16]. However, as mentioned above, ICP-AES and ICP-MS need relatively complicated pretreatment processes and, therefore, real-time analysis is difficult. Additionally, the large-scale and high-cost equipment that is necessary for those analysis techniques is not cost-effective for most healthcare applications. Therefore, many groups have proposed LIBS techniques for the analysis of biological samples. LIBS spectra of fingernails have been measured for the diagnosis of diseases, such as diabetes. As nails are relatively hard tissues with high mechanical and chemical strength, stable LIBS measurements are possible [17,18,19,20].

Hair is another target tissue for LIBS measurements because the concentrations of trace elements in hair are generally higher than that in other biological tissues. Haruna detected Ca in human hair using LIBS and qualitatively analyzed Ca variation with age and sex [21]. Corsi et al. quantitatively analyzed mineral content (Mg, K, Ca, Na, and Al) in human hair and compared their results with those obtained through a commercial analytical laboratory [22]. Emara measured trace elements in horsehair and compared the results with those obtained via atomic absorption spectroscopy [23]. More recently, Zhang combined LIBS with ultrasound-assisted alkali dissolution to analyze Zn and Cu more accurately in human hair [24]. In these LIBS applications, conventional Q-switched Nd: YAG lasers are used. This is because conventional Q-switched Nd: YAG lasers can obtain relatively high plasma intensity by irradiation with pulse energy of more than 10 mJ, which is necessary to detect trace elements in hair. The use of these lasers makes the LIBS system large in scale and not easy to handle. In addition, irradiation with high energy pulses easily induces severe damage to hair and sometimes the hair is torn off, which can cause changes in the detected signal while obtaining the LIBS spectra.

In this paper, we propose LIBS analysis of human hair while using a microchip laser for plasma generation. To obtain plasma intensity that is sufficiently high for trace element analysis, we optimally designed the focusing optics. We then accumulate hundreds of LIBS spectra generated by the microchip laser emitting at high repetition rate pulses while Argon gas is injected at the irradiation point to enhance plasma intensity. Table 1 presents the typical concentrations of trace elements contained in the black hair of Japanese adults [25]. Among these elements, we analyzed the relative mass concentrations of Ca, Mg, and Zn of hairs obtained from five subjects while using the intensity of C as a reference. The concentration results coincide well with those measured via ICP-MS.

## 2. Materials and Methods

**Figure 1:**
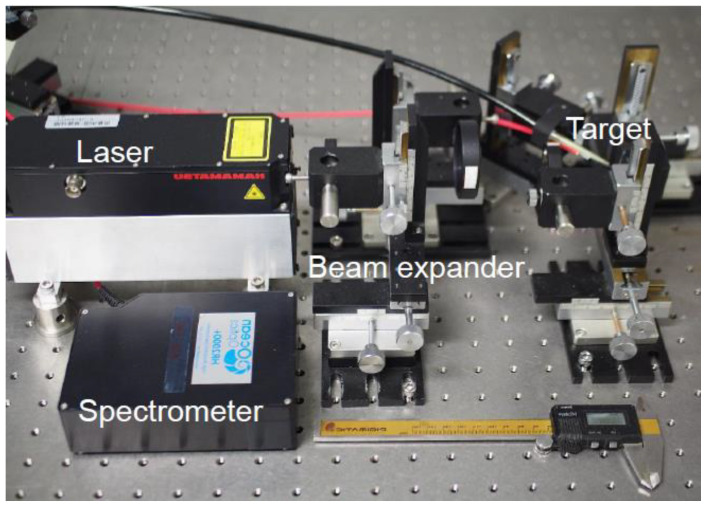
Measurement setup.

In our experiment, a passively Q-switched microchip laser (L11038–11, Hamamatsu Photonics, Hamamatsu, Japan) emitting optical pulses with an energy of 2 mJ and pulse duration of 1 ns at a wavelength of 1064 nm was used as the light source. The measurement setup is shown in Figure 1. The experiment was conducted under atmospheric pressure. The plasma generated from the sample was delivered to the spectrometer by a step-index silica-glass fiber with a core diameter of 600 µm and NA of 0.22. The distal end of the fiber was located at a distance of 2 mm from the irradiation point. The spectra were measured using a fiber-coupled spectrometer (HR2000+, Ocean Insight, Orlando, FL, USA) which was synchronized with the laser pulses using an external pulse generator. The integration time of the spectrometer was set to 1 ms, which was the shortest possible setting, and was not gated. Therefore, the plasma emission generated by a nanosecond optical pulse was completely detected. Three types of spectrometers were used to obtain LIBS spectra from the ultraviolet (UV) to visible (Vis) regions. With the combined use of three spectrometers, the wavelength ranges of 200–343 nm, 355–474 nm, and 480–597 nm were covered with a wavelength resolution of 0.14 nm.

Dozens of human hairs were bundled and fixed on a metal substrate to create samples. The substrate had a hole with a diameter of 12 mm at the center to avoid generating the plasma from the underlying substrate. The hairs were washed twice with pure water after degreasing with acetone. When irradiating with optical pulses, the sample was moved slowly along the longitudinal direction at a speed of approximately 2 cm/min to avoid severely damaging the hair sample. Our protocol was approved by the ethical committee on the Use of Humans as Experimental Subjects of Tohoku University (No. 20A-29), and informed consent was obtained from the examinees.

**Figure 2:**
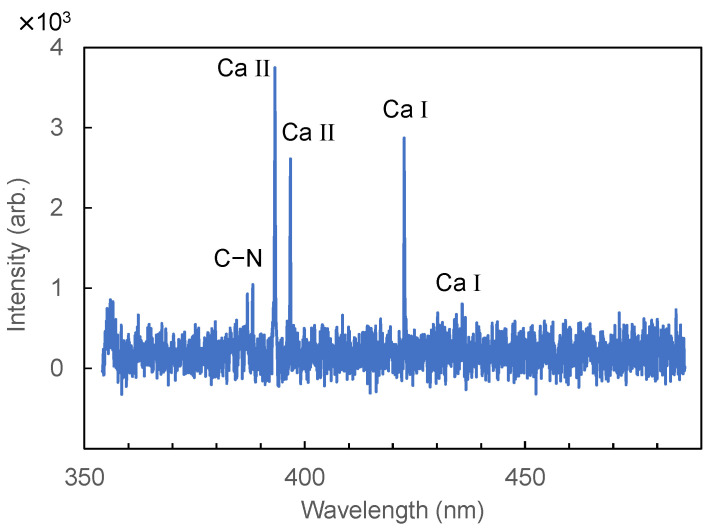
Laser-induced breakdown spectroscopy (LIBS) spectrum of hair measured by a focusing system with a single lens.

We first focused the laser beam, using a spherical BK7 lens with a focal length of 100 mm, to obtain the LIBS spectrum shown in Figure 2. For each measurement, 300 consecutive spectra were accumulated. Because of the limited data acquisition speed of the spectrometer, the repetition rate of the laser was set to 10 Hz, and thus, it took 30 s for a single measurement. To identify elements from the LIBS spectra, we referred to the NIST Database [26] and OSCAR Database [27]. Although we confirmed emission peaks of Ca at Ca I: 422.7 and 445.5 nm, Ca II: 393.3 and 396.8 nm, and of the C–N bond at 388.29 nm, we could not detect peaks of another material because of the low signal-to-noise ratio (SNR) of the LIBS system.

**Figure 3:**
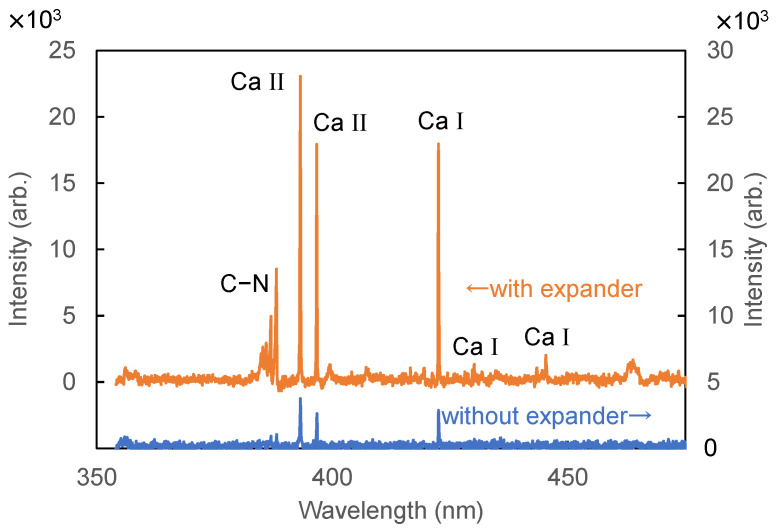
LIBS spectra of hair measured with and without an expander.

To improve the SNR, we designed focusing optics to increase the optical power density at the focusing spot. To obtain a more focused beam spot, we built a Galilean beam expander with 5× magnification. The focused beam size was reduced to 0.12 mm from 0.37 mm by introducing the expander. The power density at the focal spot was approximately 4.5 × 10^9^ W/cm^2^. Figure 3 shows a LIBS spectrum of hair measured with and without the beam expander. We found that the peak intensity was enhanced by approximately 10 times.

**Figure 4:**
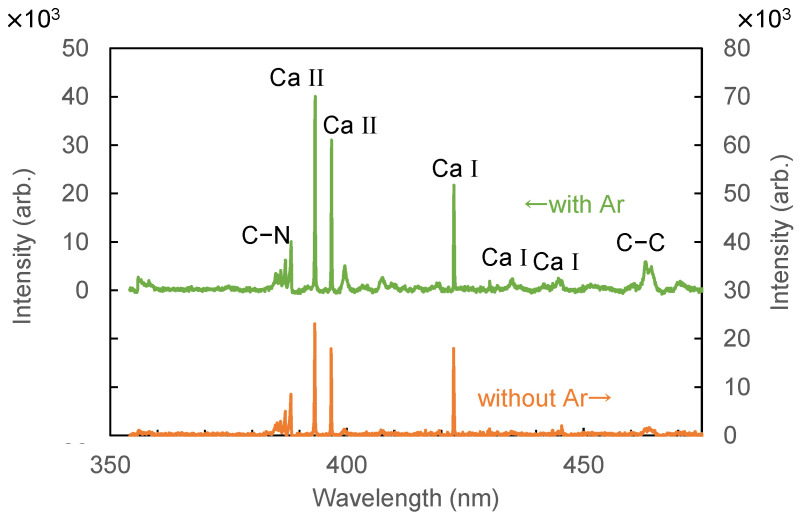
LIBS spectra of hair measured with and without argon gas injection.

To further increase the sensitivity, we injected argon gas onto the focusing spot. It was reported that the optical emission intensity of plasma induced by laser irradiation is enhanced in the argon atmosphere [28]. One of the reasons for this is that the energy decay of free electrons in plasma is suppressed in an argon atmosphere. In addition, high plasma temperature is maintained in argon because of its lower thermal conductivity. Figure 4 shows LIBS spectra of human hair measured with and without argon injection. Argon gas was injected from a nozzle set at a distance of 2 mm from the focused spot with a flow rate of 1 L/min. It was found that the peak intensity was enhanced approximately 1.8 times using argon injection; small peaks that can be attributed to C–N bond were observed at around 358 nm.

**Figure 5:**
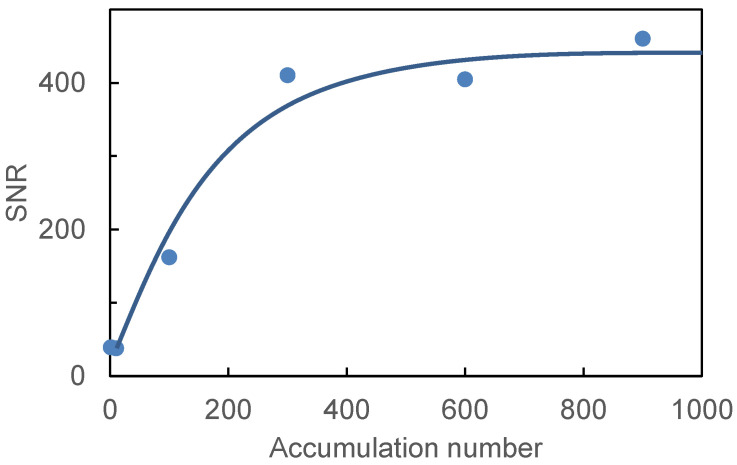
Correlation between signal-to-noise ratio (SNR) of obtained spectra and accumulation number.

To find the optimum number of spectrum accumulation in a LIBS measurement, we changed the accumulation number from 1 to 900 and measured the LIBS spectra of human hair. Figure 5 shows the correlation between the SNR of the obtained spectra and the accumulation number. The SNR was defined as (Peak area of Ca II at 393.3 nm)/[(standard deviation of background signal) × (full width of the Ca II peak at half maximum)]. We found that the SNR was almost saturated at an accumulation number of 300, and therefore, we set the accumulation number to 300 in consideration of the measurement time.

## 3. Results and Discussions

**Figure 6:**
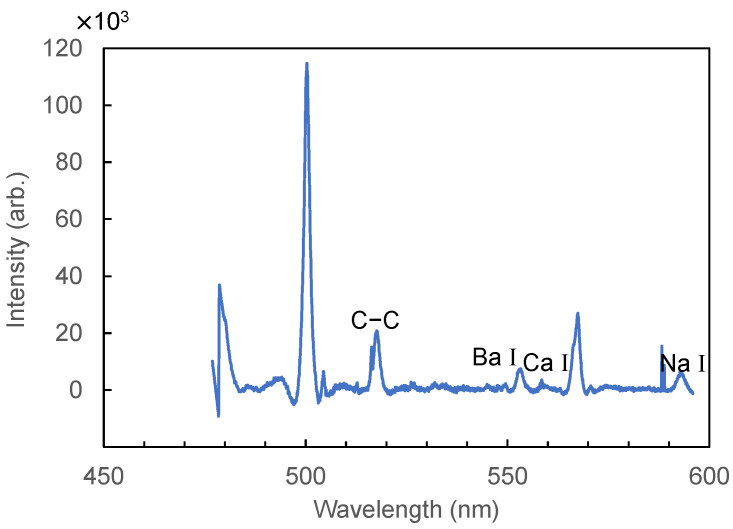
LIBS spectrum of hair measured in the visible wavelength region.

**Figure 7:**
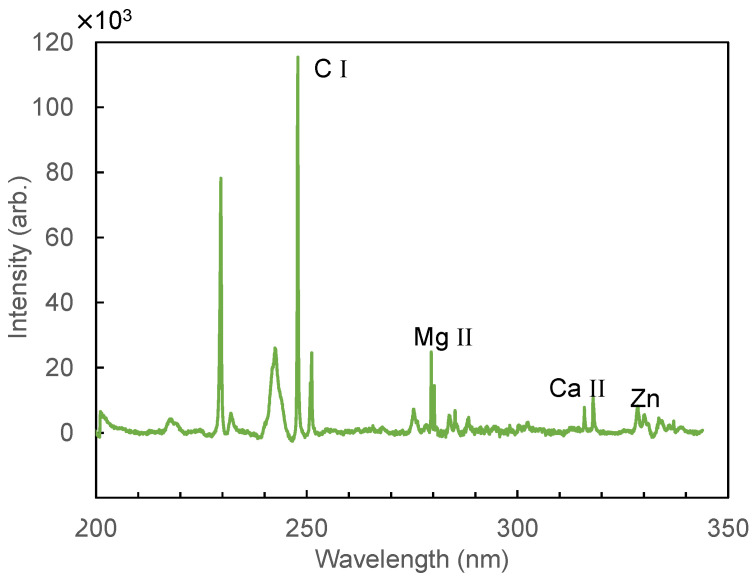
LIBS spectrum of hair measured in the ultraviolet wavelength region.

**Figure 8:**
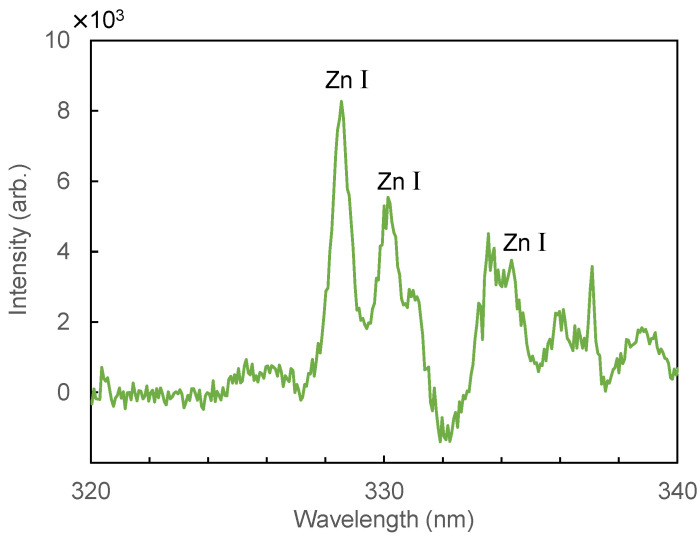
An enlarged LIBS spectrum of hair measured at around 330 nm.

Figure 6 shows a LIBS spectrum of human hair measured from 480 to 597 nm. In this wavelength region, emission peaks for the C–C bond at 512.8 and 516.4 nm, Ba I at 553.5 nm, and Na I at 589.6 nm are observed. Figure 7 shows a spectrum measured at 200–340 nm. In the measurement results for the UV region, we found that there was less background noise from calcium emission; thus, we changed the focal length of the lens from 100 mm to 50 mm to further increase the energy density at the focused spot. The focal spot size was reduced to 0.09 mm from 0.12 mm. In this region, we observed peaks of C I (247.8 nm), Mg II (279.5 nm), and Ca II (315.8 nm). Figure 8 shows an enlarged spectrum at around 330 nm. Although the peak intensities are relatively low, we found that the peaks coincide with that of Zn I at 328.26, 330.26, and 334.51 nm. Since it was found that the peak at 330.26 nm was affected by the peak of Na I at 330.3 nm, we hereafter use the peak at 328.26 nm for analysis of Zn I, which is one of the trace minerals essential for human life.

We performed LIBS measurements to observe individual differences in the relative concentrations of the trace minerals. We collected hair samples from five volunteers, aged 23–25, and compared the results of LIBS analysis to those obtained via ICP-MS analysis. LIBS measurements were used to observe individual differences in the relative concentrations of these trace minerals. As the concentration of trace elements in hair depends on the position along the hair length, we analyzed the hair around 2.5 cm from the root for all the measurements. We utilized the commercial service of the Kyorin Preventive Medicine Institute [29] for ICP-MS analysis of hair samples that were taken from the same subjects at the same time. For ICP-MS analysis, the same part of hair as described above was used for comparisons.

As the intensity of observed peaks varies widely because of the small diameter of the hair samples, the relative concentrations obtained via LIBS analysis were calculated by setting the peak intensity of C I as the reference because C is a primary composition component of hair; as such, the individual difference should be relatively small. Table 2 shows the observed peak intensities of Mg II (279.5 nm), Zn I (328.26), and Ca II (315.8 nm) measured for samples taken from the five subjects. The coefficients of variation (CV) were calculated from the results of three measurements for each subject, and we confirmed that the variations reduced considerably upon using the peak intensity of C as a reference. We also tried to normalize the peak intensities by the total emission intensity of measured LIBS spectra. However, we did not obtain better results because of the relatively large background noises in the measurement. The calculated total emission intensity largely changed with baseline correction processing.

**Figure 9:**
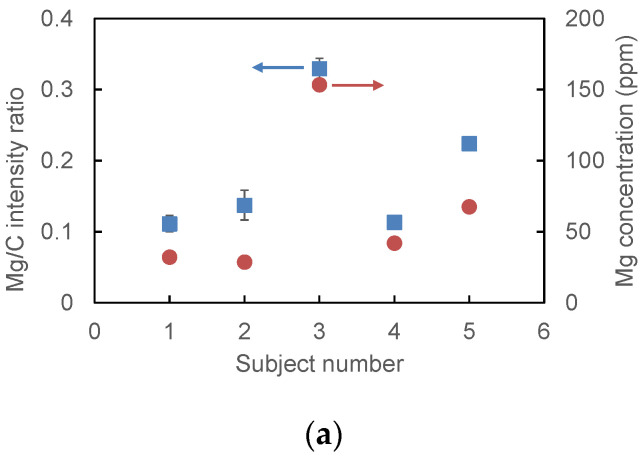

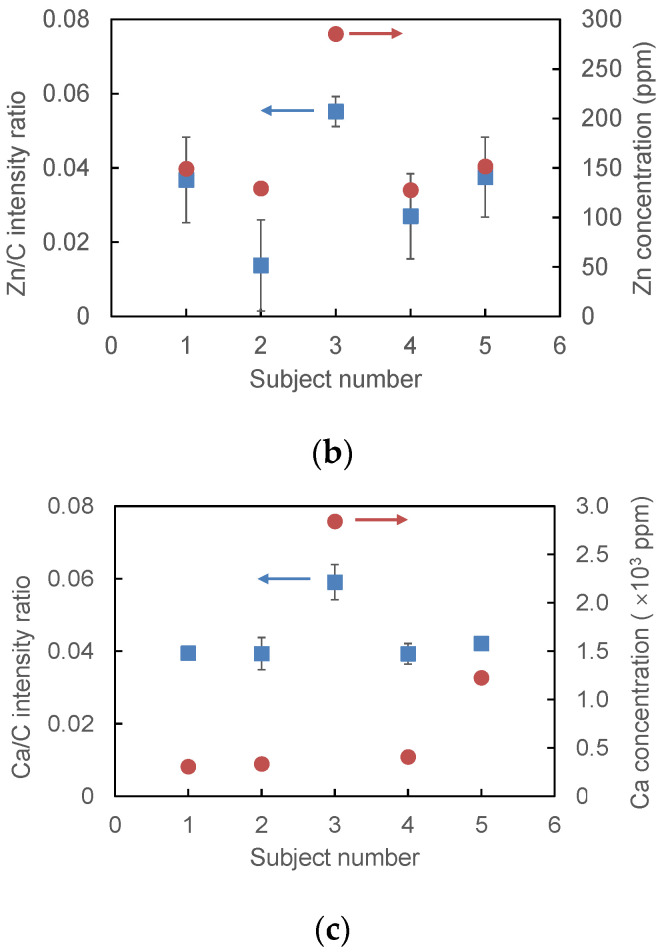
Relative mass concentrations of (**a**) Mg, (**b**) Zn, and (**c**) Ca measured for the five subjects and compared with the absolute concentrations analyzed via ICP-MS.

Figure 9 shows the relative mass concentrations of Mg, Zn, and Ca measured for the five subjects compared with the absolute concentrations analyzed via ICP-MS. In the LIBS results in Figure 9, the dots are the average values of three measurements, and the error bars show the minimum and the maximum measured values. The measurement variability was sufficiently small to see individual differences and the trends between the subjects coincided with the results of ICP-MS analysis.

We confirmed good linearity between the results of the LIBS and ICP-MS methods, and the determination coefficient R^2^ was 0.921 for Mg, 0.670 for Zn, and 0.952 for Ca. We observed relatively small correlation for Zn; this may be because of the small peak intensity compared to the ones of Mg and Ca. The lowest detectable concentrations, defined by SNR = 3, were 9.0 ppm for Mg, 27 ppm for Zn, and 710 ppm for Ca. Since these values are lower than typical concentrations of these trace minerals, we confirmed the feasibility of the proposed LIBS using a microchip laser for analysis of relative mass concentrations in human hair. In the above analysis, we did not consider the variability of plasma temperature, electron density, and upper energy levels of the observed transitions [30]. For more accurate analysis of trace elements in human hair, corrections based on these factors may be necessary.

## 4. Conclusions

As a compact and low-cost LIBS system for in-situ analysis of trace minerals in human hair, we proposed a system using a microchip laser for plasma generation. Since the pulse energy emitted from a microchip laser is lower than that of conventional flashlamp-excited Q-switched Nd:YAG lasers, we optimally designed the focusing optics utilizing a Galilean beam expander to obtain plasma intensity sufficiently high for analysis of trace elements in hair. Additionally, we accumulated hundreds of LIBS spectra to improve the SNR of the measurement system and injected argon gas on the irradiation point to enhance the plasma intensity. After investigating LIBS spectra of human hair in the UV to near IR regions, we focused on the spectra in the UV region because of the location of emission peaks of Mg and Zn, which are trace minerals essential for human life.

We analyzed relative mass concentrations of Ca, Mg, and Zn in hairs obtained from five subjects while using the intensity of the C peak as a reference. The results coincided well with those measured via ICP-MS. We estimated the lowest detectable concentrations from the SNR of the measured LIBS spectra: 9.0 ppm for Mg, 27 ppm for Zn, and 710 ppm for Ca. From these results, we have concluded that the proposed LIBS system based on a microchip laser is feasible for the analysis of trace minerals in human hair.

Owing to the low-cost and compact proposed system, we expect that it will be a useful biomedical sensor for health-care applications based on non-invasive and real time analysis of hair.

## Figures and Tables

**Table 1:** Typical concentrations of trace elements contained in the black hair of Japanese adults [25].

Element	Ca	Zn	Mg	Fe	Al	K	Na
Concentration (ppm)	810	179	164	144	130	70	65

**Table 2:** Effect of normalization based on the peak intensity of C I at 247.8 nm. In the table, “X” and “X/C” show the coefficients of variations (CV) before and after normalization, respectively.

Subject		1			2			#3			4			5		
Element X		Mg	Zn	Ca	Mg	Zn	Ca	Mg	Zn	Ca	Mg	Zn	Ca	Mg	Zn	Ca
CV	X	0.126	0.350	0.134	0.159	0386	0.209	0.249	0.573	0.285	0.034	0.053	0.141	0.107	0.151	0.157
	X/C	0.043	0.189	0.037	0.102	0.212	0.046	0.152	0.505	0.055	0.044	0.022	0.082	0.023	0.170	0.073

## Data Availability

The data presented in this study are available on request from the corresponding author. The data are not publicly available due to privacy restrictions.
